# Natural Knockouts: Natural Selection Knocked Out

**DOI:** 10.3390/biology6040043

**Published:** 2017-12-12

**Authors:** Peter Borger

**Affiliations:** The Independent Research Initiative on Information & Origins, 79540 Loerrach, Germany; peterborger@hotmail.com

**Keywords:** genetic redundancy, natural knockout, natural selection, homozygous loss-of-function

## Abstract

In functional genomics studies, research is dedicated to unveiling the function of genes using gene-knockouts, model organisms in which a gene is artificially inactivated. The idea is that, by knocking out the gene, the provoked phenotype would inform us about the function of the gene. Still, the function of many genes cannot be elucidated, because disruption of conserved sequences, including protein-coding genes, often does not directly affect the phenotype. Since the phenomenon was first observed in the early nineties of the last century, these so-called ‘no-phenotype knockouts’ have met with great skepticism and resistance by died-in-the-wool selectionists. Still, functional genomics of the late 20th and early 21st centuries has taught us two important lessons. First, two or more unrelated genes can often substitute for each other; and second, some genes are only present in the genome in a silent state. In the laboratory, the disruption of such genes does not negatively influence reproductive success, and does not show measurable fitness effects of the species. The genes are redundant. Genetic redundancy, one of the big surprises of modern biology, can thus be defined as the condition in which the inactivation of a gene is selectively neutral. The no-phenotype knockout is not just a freak of the laboratory. Genetic variants known as homozygous loss-of-function (HLOF) variants are of considerable scientific and clinical interest, as they represent experiments of nature qualifying as “natural knockouts”. Such natural knockouts challenge the conventional NeoDarwinian appraisal that genetic information is the result of natural selection acting on random genetic variation.

## 1. Natural Knockouts

In vertebrates, angiogenesis is an intricate process involving many cooperating genes, proteins and regulatory molecules. One of them is angiogenin. It is a small protein that stimulates blood vessel formation, and can be found in all vertebrates, including humans and mice. In cancer research, angiogenin has been the topic of extensive scrutiny, since tumors produce blood vessel promoting growth factors, including angiogenin. In order to grow and expand, tumors need food and oxygen, which are delivered via blood vessels. The idea was that, if the supply of angiogenin could be reduced, the tumor would be unable to form vessels and it would die because of lack of oxygen and nutrients. Hence, targeting angiogenin became a major topic of research that held the promise to reduce the growth of tumors. To elucidate how angiogenin functions in humans, functional genomics usually turns to a mouse model, in which the gene of interest is interrupted and not expressed. Over the years, thousands of knockout models have been generated, and the results have significantly contributed to our understanding of how single-copy genes function in real-life systems. Conceivably, cancer researchers would like to have a mouse knockout for the angiogenin gene. Unfortunately, the genome of the mouse contains three copies of the angiogenin gene. Three copies of the same gene form a natural back up. Disrupting one of them will hardly give an informative phenotype, since the two remaining copies would still be active and able to produce the functional angiogenin protein. To create an informative angiogenin knockout mouse model for research purposes, three copies of angiogenin have to be interrupted all at once—and that is very hard to achieve. The haploid genomes of primates only contain one copy of the angiogenin gene, and therefore would make better angiogenin knockout models. Interestingly, this model is present in nature. The Douc Langur (*Pygathrix nemaeus*) is an Asian leaf-eating Colobine monkey, and is the natural knockout for angiogenin. The Douc Langur does not have a functional angiogenin protein, because the gene contains a one-nucleotide deletion mutation in the sixth codon. The deletion induces a frame shift leading to a premature stop codon, so that only truncated angiogenin proteins that do not show biological function are produced. The investigators who reported this natural knockout for angiogenin concluded that primate angiogenin is dispensable—even in natural populations [1]. These in vivo observations demonstrate that the angiogenin gene qualifies as a redundant gene. How can single-copy genes be dispensable in a NeoDarwinian world? How could angiogenin be naturally selected if an operable blood vessel formation system was already in place? What was the selective constraint that prompted the angiogenin gene to evolve?

## 2. The CCR5 Entry

Acquired immunodeficiency syndrome (AIDS) is caused by the human immunodeficiency virus (HIV). A world-wide pest, the disease impacts the life of millions of people, yet not all humans are equally vulnerable to HIV infections. A considerable part of the European population seems to be completely resistant to HIV infections, or are at least to some extent protected [2]. The origin of this peculiar immunity can be found in the genetic make-up of millions of Europeans. The protected subjects have a deletion in the gene that specifies a chemokine receptor known as CCR5. In Caucasians, the frequency of this deletion, known as the CCR5 delta32 allele, is 10–20 percent, and the prevalence of the homozygous mutation is 1–2 percent [2]. The CCR5 receptor, which is located on the cell surface, can be exploited by viruses to gain entry into CCR5 expressing cells. HIV uses the CCR5 receptor IVHI to enter T cells and macrophages, two pivotal cell types of the immune system, which help it to mount an appropriate immune response to microscopic intruders. As soon as HIV has entered these cells, it hijacks its biochemistry and genetic apparatus, and redirects them to the benefit of its own reproduction. Normally, this leads to the obliteration of the hijacked immune cells. The mutation that protects a subset of the European population causes a truncated CCR5 receptor, thereby rendering it non-functional. Individuals who have inherited the mutation from both of their parents, the homozygous loss-of-function (HLOF) variants, qualify as human natural knockouts. Since they completely lack the CCR5 on their cell surfaces, the AIDS-causing virus cannot enter the immune cells. As a result, the CCR5 knockouts that we observe in the European population are entirely or partially resistant to HIV infections. The individuals that inherited an inactivated CCR5 gene from only one of their parents still have one functional copy, but now HIV has more trouble entering the cells. AIDS is a deadly disease. People lacking both functional CCR5 copies—the natural knockouts—should be under intense natural selection in populations with a high prevalence of HIV. Why do we observe the chopped-off-receptor in a huge part of the European population? HIV is a very new virus; most studies showed the virus originated in Africa between fifty and one hundred years ago, and cannot have killed Europeans long enough for natural selection to have favored it. Therefore, it has been hypothesized that other epidemic-causing viruses, in particular smallpox, may have increased the prevalence of the truncated CCR5 gene in medieval Europe [2]. The geographical distribution of the inactive gene indeed demonstrate that the average frequency of the truncated CCR5 gene is around 10 percent across Europe, with high incidences of over 15 percent in Iceland and the Baltic countries, and 4 percent in the Sardinian population [2]. Interesting, humans are not the only HLOF mutants for CCR5. They are also observed in an African rainforest primate species known as the red-capped Mangabey (*Cercocebus torquatus*). The primate is the natural host of simian immunodeficiency virus (SIV), a virus very similar to HIV, which also requires the CCR5 receptor to gain entry to cells. Red-capped Mangabeys likewise lack a fragment of the CCR5 gene, a 24-base pair deletion, which also prevents the expression of a functional CCR5 protein at the cell surface, and thus has a similar immunizing effect as the truncated CCR5 gene in Europeans [3]. Millions of Europeans and almost the whole population of African Mangabeys are natural knockouts for CCR5.

## 3. The EPO Switch

In 1964, Eero Maentyranta, a Finnish cross-country skier, won two gold medals during the Winter Olympics in Innsbruck. His 15 and 30 km skiing successes were controversially debated, however, because blood tests showed he had 15 percent more erythrocytes than the other winter athletes had. Not surprisingly, Maentyranta was suspected of using blood doping, although all laboratory tests to identify possible stamina-increasing compounds were negative. Since no trace of blood doping could be found, the skier returned to Finland with two gold medals. In 1964 nobody knew, but modern molecular biology research showed that Maentyranta has a mutated *EPO* gene that increases red blood cell production through high levels of *erythropoietin*. To increase red blood levels, EPO binds to a receptor present on uncommitted stem cells in the bone marrow that then develop into red blood cells. The receptor is a membrane-bound protein with several domains that fulfill distinct functions. Upon EPO binding, one of the domains generates a signal to instruct bone marrow cells to become red blood cells. This is the on-switch of the EPO receptor. Another domain of the receptor acts as an off-switch, and tells the stem cells to stop producing red blood cells. This auto-regulatory mechanism assures that red cell counts never exceed physiological boundaries. In 1993, geneticists determined that the Olympic medalist has a deletion at the far end of the gene coding for the EPO receptor [4]. To be precise, the mutation chops off the final 70 amino acids of the domain that normally forms the off-switch. The off-switch is known as the docking site for *shp*, a negative regulatory protein that terminates the signal. Binding of erythropoietin its receptor not only generates a signal that communicates the cells to differentiate in order to spawn more red blood cells. It also changes the shape of the receptor in such way that phosphate groups can be added to newly exposed domains where *shp* can dock and turn off the signal by removing the phosphate groups added by EPO. The result is that receptor is transformed back to its original, inactive state. *Shp* proteins are always present, and thus are always available to terminate the signal delivered by EPO. As soon as the switch is turned on, *shp* turns it off in an almost instant event. Maentyranta did not have to take blood doping or exercise at high altitudes; high levels of red blood cells run in the family. The mutated receptors in the Finnish athlete generated a normal activation signal, but not the deactivation. In the Maentyranta family, the production of red blood cells in the bone marrow is out of control because the off-switch broke off. Even low levels of EPO cause the red cell progenitors to grow and divide. Production of red blood cells simply cannot be stopped! The profound excess of red blood cells observed in the athlete may explain why Maentyranta had more stamina than his competitors. Missing the negative feedback domain of the EPO receptor does not seem to affect the fitness of the carrier, who thus qualifies as a natural knockout.

## 4. The Endurance Gene?

Alpha-actinins are structural proteins of muscle fibers. They anchor the thin filaments and maintain the spatial relationship between the tiny fiber units that make up the muscles—the myofilaments. In humans, two types of alpha actinin genes exist (*ACTN2* and *ACTN3*), which encode two closely related proteins. *Alpha-actinin-2* is expressed in all skeletal muscle fibers, whereas *alpha-actinin-3* is limited to a subset of very fast muscle fibres, the so-called fast-twitch muscles. The proteins have been the subject of considerable interest as they could potentially play a role in muscle abnormalities. A group of Australian researchers carefully screened muscle tissue from individuals with dystrophic, myopathic, neurogenic muscle disorders and compared the results with normal subjects. All biopsies expressed a functional alpha-actinin-2 gene. Unexpectedly, a surprising 20 percent of the biopsies did not contain a functional alpha-actinin-3 gene. The deficiency turned out to result from a common *nonsense mutation*, which results in a truncated defunct alpha-actinin-3 protein. The study showed that the absence of a functional alpha-actinin-3 gene is *not associated* with any particular pathological or clinical phenotype [5]. In humans, the alpha-actinin-3 gene can be missed entirely, and natural knockouts abound. The mutation that inactivated the *ACTN3* gene was detected in all studied human populations—including the indigenous peoples of Australia, Africa and America [6]. Because the absence of the gene does not result into diseases and does not seem to affect fitness, the *ACTN3* gene is redundant in humans. Interestingly, a significantly higher number of sprinters have two normal copies of the *ACTN3* gene, while among endurance athletes, two inactive copies is more common [7]. Still, the natural knockout for *ACTN3* is also present in the population of sprinters, indicating that sprinters do not require an active *ACTN3* gene [8]. This is a very revealing observation. If a population of sprinters can do without the *ACTN3* gene, *the gene is redundant,* and we have identified another natural knockout in the healthy, non-diseased human population. Other studies have revealed healthy natural knockouts for Glutathione-S Transferase genes (GSTM1 and GSTT1) that play a role in the detoxification process of polycyclic aromatic hydrocarbons present in tobacco smoke [9].

## 5. Each of Us: A Natural Knockout for Many Genes

Fascinated by the idea that hidden in the human population there are individuals who lack certain genes, yet remain entirely healthy, Daniel MacArthur, one of the investigators of the *ACTN3* work, started a survey of scores of human genomes in search of additional missing genes. After scanning 185 individual genomes, he and coworkers showed that the average healthy person carries about 100 debilitated genes, with 20 of those being present in a homozygous state [10]. Every individual in the human population is a natural knockout for 20 protein-coding genes! Several of the missing genes played a role in the olfactory system. Others were members of functionally overlapping gene families, suggesting the inactivated genes were lost because a backup existed elsewhere in the genome. In the set of debilitated genes, 24 are known to be involved in atrocious diseases, including *osteogenesis imperfecta* and *harlequin ichthyosis*, as well as 21 disease-causing genes—but these were all found in only one copy in the affected individuals [11]. The majority of the high-confidence HLOF variants were found in less than 2% of the population. The non-diseased phenotypes of many HLOFs demonstrate that natural selection is unable to preserve these genes, cannot remove debilitating mutations, and so, one after the other, the genes are inactivated. More recently, using 1432 whole exome sequences from five European populations, the catalogue of known human HLOF mutations further expanded; after stringent filtering, a total of 173 HLOF mutations, 76 (44%) of which had not been observed previously, were identified [12]. The Exome Aggregation Consortium (ExAC), which has generated the largest database of variation in human protein-coding regions, aggregated sequence data from more than 60,000 people [13], which are publicly accessible. Using this catalogue to calculate objective metrics of pathogenicity for sequence variants, and to identify genes subject to strong selection against various classes of mutation, only 3230 genes with near-complete depletion of predicted protein-truncating variants were identified [14]. In other words, most protein-coding genes in the human genome can be found inactive in one or more individuals.

## 6. Selection’s Inaptness

The Natural Knockout is a phenomenon not only found in primates. In natural populations of *Arabidopsis thaliana,* it was observed that almost every tenth gene was so defective that it could not fulfill its normal function anymore [15]. The natural knockout seems the norm, and may be explained by the fact that the biological information of the genomes of sexually reproducing individuals is always present 2-fold. After fertilization, all genes present in the nucleus are backed up by at least one additional copy, providing 2-fold redundancy of the entire genome. Sexual reproduction thus provides individuals genetic robustness, since corruptive mutations in essential genes would not immediately harm the organism’s fitness. Still, 2-fold redundant genomes will accumulate corrupted genes in a heterozygous state. When two individuals with the same corrupted genes mate, part of the offspring will be homozygous for the corrupted genes: HLOF. Considering the current data, HLOF is an often-observed phenomenon—and HLOF has recurrently failed to yield diseased phenotypes. Adaptations associated with HLOF are then solely due to loss of genetic information.

A model of genetic redundancy due to gene duplications demonstrated that the redundancy can only be maintained by selection when populations are large (such as bacteria), or when mutation rates are high. On average, mutation will always lead to decreased redundancy [16]. Further studies showed that weak or no-effect genes (i.e., non-essential and redundant genes) are no more likely to have paralogous genes than genes that do result in a defined phenotype when they are knocked out [17,18]. Hence, gene duplication is not the cause of redundancy, which is, rather, due to unrelated genes with the same or very similar function. Although the 2-fold redundancy (diploidy) of sexually reproducing organisms is an unexplained fact of nature (the origin of sex is unknown), it may exist to stabilize the phenotype to produce more robust organism—and to prevent evolutionary changes. Nevertheless, natural selection cannot stop the decline of redundant genetic information. Without additional genome-stabilizing mechanisms, save natural selection, the genomes of diploid organisms are bound to disintegrate.
